# The Fate of Chrysotile-Induced Multipolar Mitosis and Aneuploid Population in Cultured Lung Cancer Cells

**DOI:** 10.1371/journal.pone.0018600

**Published:** 2011-04-05

**Authors:** Beatriz de Araujo Cortez, Gonzalo Quassollo, Alfredo Caceres, Glaucia Maria Machado-Santelli

**Affiliations:** 1 Departamento Biologia Celular e do Desenvolvimento, Instituto de Ciências Biomédicas, Universidade de São Paulo, São Paulo, Brasil; 2 Departamento Genética e Biologia Evolutiva, Instituto de Biociências, Universidade de São Paulo, São Paulo, Brasil; 3 Instituto de Investigación Médica Mercedes y Martín Ferreyra (INIMEC-CONICET), Córdoba, Argentina; The Hong Kong University of Science and Technology, Hong Kong

## Abstract

Chrysotile is one of the six types of asbestos, and it is the only one that can still be commercialized in many countries. Exposure to other types of asbestos has been associated with serious diseases, such as lung carcinomas and pleural mesotheliomas. The association of chrysotile exposure with disease is controversial. However, *in vitro* studies show the mutagenic potential of chrysotile, which can induce DNA and cell damage. The present work aimed to analyze alterations in lung small cell carcinoma cultures after 48 h of chrysotile exposure, followed by 2, 4 and 8 days of recovery in fiber-free culture medium. Some alterations, such as aneuploid cell formation, increased number of cells in G2/M phase and cells in multipolar mitosis were observed even after 8 days of recovery. The presence of chrysotile fibers in the cell cultures was detected and cell morphology was observed by laser scanning confocal microscopy. After 4 and 8 days of recovery, only a few chrysotile fragments were present in some cells, and the cellular morphology was similar to that of control cells. Cells transfected with the GFP-tagged α-tubulin plasmid were treated with chrysotile for 24 or 48 h and cells in multipolar mitosis were observed by time-lapse microscopy. Fates of these cells were established: retention in metaphase, cell death, progression through M phase generating more than two daughter cells or cell fusion during telophase or cytokinesis. Some of them were related to the formation of aneuploid cells and cells with abnormal number of centrosomes.

## Introduction

Asbestos is a silicate mineral divided in two major groups - serpentines and amphiboles. Amphibole fibers were commonly used in commercial applications until the association of amphibole exposure with several serious diseases, such as asbestosis, bronchial cancer and malignant mesothelioma of the pleura and peritoneum [1], [2]. Currently, amphibole fibers cannot be used in many countries and have been replaced by chrysotile, a serpentine asbestos that is considered less harmful to human health.

Chrysotile is composed of curved silken fibers with a small transverse section (80 to 130 Å) and a tubular structure. Its clearance from lung tissue is faster than that of amphibole fibers, and chrysotile does not accumulate in the lung due to a mechanism involving fragmentation of the fibers into short pieces [3].

The mechanisms leading to the development of diseases like carcinomas and mesotheliomas after asbestos exposure are not well understood. However, the mutagenic and cytotoxic effects of asbestos have been shown in studies using cultured cells exposed to different asbestos fibers for periods ranging from 1 h to 72 h.

Exposure of cultured cells to asbestos leads to the formation of oxyradicals and free radicals that damage DNA [4], [5], [6]. It has been shown that exposure of cultured cells to chrysotile can cause double strand breaks in DNA after 3 and 24 h [7], [8] and can also cause intrachromosomal deletions and DNA mutations [9]. Chrysotile-induced DNA damage can trigger apoptosis in various cell types following 3 to 4 h of exposure [8], [10].

Micronuclei are also observed after chrysotile treatment [11]. These chromatin bodies, which contain an acentric chromosome fragment or an entire chromosome that detached from the metaphase plate, can be generated after chromosome breakage and mitotic disruptions, such as multipolar spindles. Therefore, the data suggest that chrysotile can cause DNA strand breaks and disrupt the mitotic spindles.

Cell cycle disruptions in cells exposed to chrysotile were investigated by flow cytometry, and complex alterations were observed. Cells treated with chrysotile for 4 to 48 h showed G2/M retention and a decreased number of S-phase cells, identified by BrdU incorporation [12].

Mitotic division following asbestos exposure was also observed by time-lapse and confocal microscopy. Some alterations were found, such as defects in spindle formation and failure of cytokinesis in the presence of internalized fibers. These experiments show that long fibers can be located between the daughter cells during telophase and lead to cytokinesis failure, generating multinucleated and aneuploid cells [13], [14]. Similar results were observed in cells exposed to carbon nanotubes. These cells showed disruptions of centrosomes and mitotic spindles, suggesting that nanotubes could interfere with microtubules and motor proteins [15].

Aneuploid cells are characterized by abnormal DNA content due to a loss or gain of whole chromosomes or parts of chromosomes, and a majority of solid tumors contain aneuploid cells [16], [17], [18]. Aneuploidy can result from errors in the cell cycle, errors in mitotic checkpoints that allow DNA damage or replication errors to be passed on to daughter cells, errors in chromosome segregation and cytokinesis, which can occur due to centrosome amplification and formation of multipolar spindles [19].

In 1914, Boveri cited aneuploidy as a cause of cancer, but the subsequent discovery of oncogenes and tumor suppressor genes led to a new theory, wherein the accumulation of mutations in such genes was the cause of malignant transformation [20]. The discussion has continued, and currently aneuploidy is considered to be involved in tumor progression, suppression or initiation [21], [22], [23]. However, it is clear that aneuploidy can introduce mutations that give rise to malignant transformation and lead to genetic instability [24].

The present work focuses on the formation of aneuploid cells after exposure to three different concentrations of chrysotile fibers, followed by long recovery times in fiber-free medium. We also analyzed cell cycle disruptions that could be involved in aneuploid cell formation, by comparing the alterations found in normal and cancerous cells exposed to chrysotile. Multipolar mitoses were also tracked to determine the fates of these cells and their contribution to aneuploid cell formation.

## Materials and Methods

### Cell Culture

The HK2 cells (a cell line established from human non-small cell lung carcinoma) [25] and VERO cells (an epithelial line derived from green monkey kidney – ATCC number CCL-81) were cultured in Dulbecco's Modified Eagle's Minimum Essential Medium (Sigma), supplemented with 10% fetal bovine serum, in a humidified atmosphere with 5% CO2 at 37°C.

### Chrysotile Treatment

Chrysotile 5R (Quebec Standard) obtained from SAMA Mineração de Amianto Ltda (Minaçu, GO, Brazil) were kindly provided by Dr. Flavia M. Cassiola. The fibers were washed with tap water and activated by sonication at controlled pH (7.4) as described elsewhere [26]. For treatment, cells were enzymatically removed from the flasks and plated in 35 mm diameter dishes (2.10^5^ cells/dish). After 24 h in culture, the medium was changed to 2 ml of fresh medium with chrysotile fibers at an approximated final concentration of 250 µg/ml, 125 µg/ml or 62.5 µg/ml, range lower than most *in vivo* studies (10 to 17 mg/m^3^). The fibers remained in contact with the cells for periods of 24 h or 48 h, after which the medium was changed. After additional periods of 2, 4 or 8 days in normal medium cells were washed with phosphate-buffered saline (PBSA) and fixed. During all the treatment the culture medium was supplemented with 10% fetal bovine serum, and changed every 2 days during recovery time.

### DNA quantification

The nuclear DNA content of chrysotile treated and control HK2 cells was quantified by image analysis with the software CIRES (Cell Image Retrieval and Evaluation System-Kontron Eletronik) installed in Axioskop microscope (Zeiss). For the analysis, the nuclei were stained by Feulgen's reaction [16]. Chrysotile treatments were done using three different fibers concentrations (250 µg/ml, 125 µg/ml, 62.5 µg/ml), and after 48 h of treatment were used three different times of recovery in fiber-free culture medium: 2, 4 and 8 days. Four hundred nuclei of mononucleated, binucleated and multinucleated were independently analyzed in control and chrysotile treated cells. Tumor cells usually have genetic alterations and most of them are hyperdiploid. Since HK2 is an *in vitro* established cell line, the diploid group was defined according to the peak of G0/G1 in the histograms, and the tetraploid group was determined with double of DNA content.

### Flow Cytometry

The cell cycle was analyzed by flow cytometry using the Guava System. Were analyzed control and chrysotile (125 µg/ml) treated cells for 48h and recovered in normal medium for 2, 4 and 8 days. For analysis cells were treated with trypsin, spun down (1,000 rpm for 10 min), washed with PBSA and fixed with methanol: PBSA (3∶1) for 1 h at 4°C. Then, cells were spun down, washed with PBSA and incubated with a solution of 200 µl of PBSA, 20 µl of RNAase and 20 µl of propidium iodide for 1 h. It was analyzed 5,000 cells for each experimental condition.

### Immunoflurescence: Mitotic Index, Cell morphology and Presence of Fibers

HK2 cells were treated with 125 µg/ml of chrysotile for 24 h and 48 h, and also treated for 48 h and recovered for 2, 4 and 8 days in normal medium; and VERO cells were treated with 125 µ/ml of chrysotile for 48 h and recovered in normal medium for 24 h. Control and treated cells were fixed with formaldehyde 3.7% for 30 min and treated with Triton X-100 0.1% for 10 min. Then the cells were submitted to immunofluorescence with anti-α and β-tubulin antibodies (Sigma, diluted 1∶200) and with the second antibody anti-mouse CY5 (Invitrogen, diluted 1∶200). After this, the cells were treated with RNAase for 30 min, the nuclei were stained by propidium iodide and the actin filaments with FITC-phalloidin. The cell morphology and presence of chrysotile fibers was imaged by laser scanning confocal microscopy (LSM 510, Carl Zeiss). These preparations were also used to quantify the presence of micronucleated, multinucleated and mitotic cells: preparations were observed by fluorescence microscopy. At least 1,000 cells/slide and 100 mitotic cells were counted in three different slides for each treatment and control.

### Time-Lapse Microscopy

HK2 were transfected with the GFP-tagget alpha-tubulin plasmid to allow the tracking of microtubules during mitosis. Transfection was performed with Lipofectamine 2000 Invitrogen) according to the manufacture protocol. After 24 h of transfection, the medium was changed to medium with chrysotile fibers. The cells remained with fibers for 24 or 48 h, and then observed by time-lapse spinning disk confocal microscopy using a DSU X-81 inverted microscope (Olympus), equipped with MT20 illumination and OSIS acquisition systems, a CCD camera (Hamamatsu, ORCA AG), and a heating chamber (Harvard Apparatus). Cell were placed on special chambers containing 2 ml of recording medium (5% Hanks balanced salt solution, 0.5% glucose, 1% fetal bovine serum, 20 mM Hepes, and 2 mM Glutamax, pH 7.3) and imaged with the DSU system using a 60x 1.42 NA oil immersion objective. Maximal projection images were obtained and processed using Metamorph software and Adobe Photoshop.

### Statistical analyses

The results were analyzed by χ2 test and P<0.05 was considered significant.

## Results

### Chrysotile concentration-dependent aneuploidy following recovery in fiber-free medium

Cultured HK2 cells were treated with three different concentrations of chrysotile, and then allowed to recover in fiber-free medium for 2, 4 and 8 days. Nuclear DNA content was quantified by image analysis and nuclei were grouped into the following five classes: hypodiploid (DNA content ≤1.49 C), diploid (DNA content between 1.5 C and 2.39 C), hyperdiploid (DNA content between 2.4 C and 3.59 C), tetraploid (DNA content between 3.6C and 5.1C) and hypertetraploid (DNA content >5.1C) (Table 1).

**Table 1:** Percentages of nuclei in hypodiploid, diploid, hyperdiploid, tetraploid and hypertetraploid classes based on DNA content.

Chrysotile concentration	Recoveryperiod	hypodiploid% (n)	diploid% (n)	hyperdiploid% (n)	tetraploid% (n)	hypertetraploid% (n)
	Control cells	3.83 (46)	58.92 (707)	20.17 (242)	16.83 (202)	0.25 (3)
**250 µg/ml**	2 days	8.83 (106)	28.75 (345)	29.08 (349)	26.27 (320)	7.4 (89)
	4 days	5.83 (70)	31.67 (380)	27.58 (331)	26.17 (314)	9.17 (110)
	8 days	5.92 (71)	31.08 (373)	29.83 (358)	22.58 (271)	10.58 (127)
**125 µg/ml**	2 days	5.50 (66)	35.08 (421)	32.17 (386)	22.75 (273)	4.5 (54)
	4 days	5.50 (66)	43.0 (516)	20.92 (251)	26.17 (314)	4.42 (53)
	8 days	4.50 (54)	53.42 (641)	16.42 (197)	20.08 (241)	5.58 (67)
**62.5 µg/ml**	2 days	8.0 (96)	44.5 (534)	26.42 (317)	17.58 (211)	3.5 (42)
	4 days	7.67 (92)	45.75 (549)	19.33 (232)	23.83 (286)	3.42 (41)
	8 days	4.50 (54)	51.17 (614)	15.42 (185)	24.25 (291)	4.67 (56)

Nuclear DNA content of control and chrysotile (250 µg/ml, 125 µg/ml or 62.5 µg/ml) -treated HK2 cells (for 48 h) allowed to recover in fiber-free medium for 2, 4 and 8 days was quantified. The nuclei were then divided into the following 5 classes: hypodiploid (DNA content ≤1.49 C), diploid (DNA content between 1.5 C and 2.39 C), hyperdiploid (DNA content between 2.4 C and 3.59 C), tetraploid (DNA content between 3.6 C and 5.1 C), and hypertetraploid (DNA content >5.1 C). For the control group and each treatment group, 1200 nuclei were analyzed. When compared to control cells, all chrysotile treatments led to hypertetraploid nuclei formation (P<0.001).

**Figure 1:**
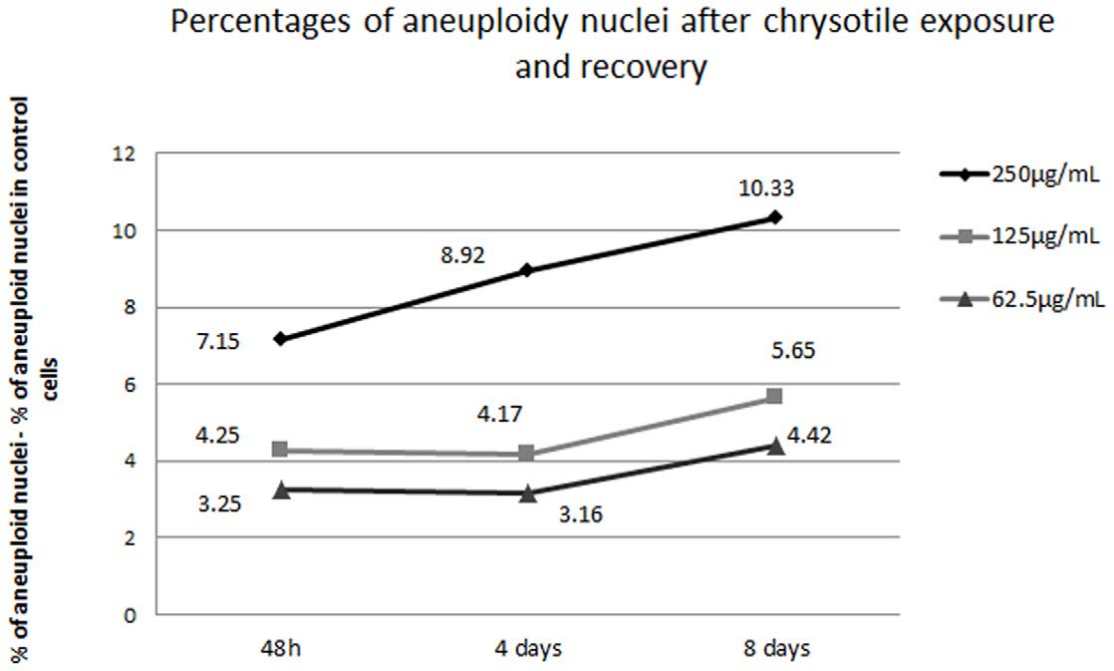
Percentage of aneuploid nuclei after chrysotile treatment and recovery. Nuclear DNA content of HK2 control and chrysotile (250 µg/ml, 125 µg/ml or 62.5 µg/ml) -treated cells (for 48 h) allowed recovering in fiber-free medium for 2, 4 or 8 days was quantified, and nuclei with DNA content >5.1 C were considered aneuploid. The percentages shown are background (the percentage of aneuploidy in control cells, 0.25%)-subtracted. Chrysotile treatment led to aneuploidy that persisted after 8 days of recovery (P<0.001).

Chrysotile treatment led to a concentration-dependent formation of hypertetraploid nuclei (also called aneuploid nuclei). After 48 h of chrysotile treatment followed by 2 days of recovery in fiber-free medium, the frequency of aneuploidy was measured. In cells treated with the highest chrysotile concentration (250 µg/mL), 7% of the cells were aneuploid, while those treated with the intermediate concentration of chrysotile (125 µg/ml) displayed 4.5% aneuploidy; the lowest chrysotile concentration (62.5 µg/ml) led to 3.5% aneuploidy, which was greater that the frequency of aneuploidy in control cells (0.25%). When analyzed after longer recovery times, the frequency of aneuploid nuclei increased, reaching 10.5% in cells treated with 250 µg/ml of chrysotile followed by 8 days of recovery. Cells treated with 125 µg/ml and 62.5 µg/ml of chrysotile and allowed to recover for 8 days presented aneuploidy rates of 5.9% and 4.67% respectively (Table 1, Fig. 1).

After 48 h of chrysotile exposure followed by 2 days of recovery, the aneuploid population was composed mostly of bi and multinuclear cells. However, after longer recovery times in fiber-free medium (4 and 8 days), aneuploid nuclei were mostly in mononuclear cells (Table 2).

**Table 2:** Percentage of aneuploid nuclei after chrysotile exposure and recovery.

Colunas1	Nuclei from mononucleated cells	Nuclei from binucleated cells	Nuclei from multinucleated cells
**Control**	100.00%	0.00%	0.00%
**250 µg/ml +2 days ^a^**	27.50%	33.75%	38.75%
**250 µg/ml +4 days ^b^**	38.10%	36.19%	25.71%
**250 µg/ml +8 days ^c^**	51.18%	28.35%	20.47%
**125 µg/ml +2 days ^d^**	31.48%	25.93%	42.59%
**125 µg/ml +4 days ^e^**	50.94%	13.21%	35.85%
**125 µg/ml +8 days ^f^**	44.78%	32.84%	22.39%
**62.5 µg/ml +2 days ^g^**	23.81%	33.33%	42.86%
**62.5 µg/ml +4 days ^h^**	41.46%	39.02%	19.51%
**62.5 µg/ml +8 days ^i^**	50.00%	25.00%	25.00%

Nuclear DNA content of control and chrysotile-treated (for 48 h) HK2 cells and allowed to recover in fiber-free medium for 2, 4 or 8 days was quantified, and nuclei with DNA content >5.1 C were considered aneuploid. Nuclei from mono-, bi- and multi-nucleated cells were quantified independently, and the aneuploid nuclei were identified. After 2 days of recovery, aneuploid nuclei were mainly in multinucleated cells, different to control cells and after long recovery periods – 4 and 8 days -, the aneuploid nuclei were predominantly in mononucleated cells. (P≤0.01: a x b, d x e, e x f, d x f; P<0.001: g x h, g x i; P = 0.1: h x i; P = 0.02: b x c).

### Cell cycle alterations after chrysotile treatment

The cell cycle in HK2 control cells and in cells treated with chrysotile for 48 h followed by 2, 4 and 8 days of recovery in fiber free medium was analyzed by flow cytometry. The treatments were performed with only one chrysotile concentration (125 µg/ml).

Cell cycle events were classified as hypodiploid/apoptotic, G1, S, G2/M and hypertetraploid cells. Similarly to the experiments with DNA quantification, the diploid group was determined according to the peak of G0/G1 cells in the histograms.

In the histograms, the first notable chrysotile-induced alteration in the cell cycle was an increase in hypodiploid/apoptotic cell formation. However, the frequency of these cells decreased after a long period of recovery, reaching the control value after 8 days of recovery (Table 3).

**Table 3:** Percentages of control and chrysotile-treated HK2 cells in the different phases of the cell cycle.

	Hypodiploid%	G1%	S%	G2/M%	Hypertetraploid%
**Control 4 days in culture**	1.55	57.17	15.36	22.56	3.36
**48 h chrysotile +2 days recovery**	3.13*	45.86*	17.13	25.55*	8.33*
**Control 6 days in culture**	2.66	56.75	16.27	21.83	2.49
**48 h chrysotile +4 days recovery**	5.55*	43.69*	16.7	26.5*	7.57*
**Control 12 days in culture**	3.21	55.95	16.53	22.21	1.87
**48 h chrysotile +8 days recovery**	4.54	46.32*	13.06	30.96*	5.12*

Cells were treated with 125 µg/ml of chrysotile for 48 h and allowed to recover in fiber-free medium for 2, 4 and 8 days, and the cell cycle was analyzed by flow cytometry. Chrysotile-treated cells showed higher numbers of G2/M and hyperdiploid cells compared with control cells in all three recovery periods. The treatment also led to lower numbers of G1 cells.

(*P<0.01).

**Figure 2:**
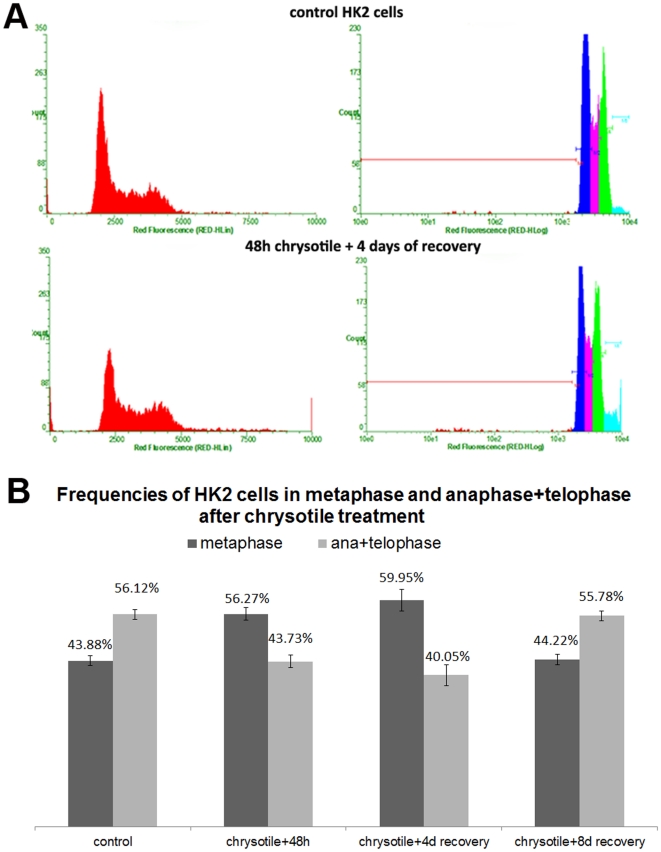
Chrysotile effects on the cell cycle and the percentage of cells in metaphase, anaphase and telophase in HK2 cells. Cells treated with chrysotile and allowed to recover for 2, 4 or 8 days in fiber-free medium were analyzed by flow cytometry and by immunofluorescence. A) Histograms in linear (red) and log (colored) scales from flow cytometry show the effects of chysotile on the cell cycle, specifically an increase in the number of G2/M and hypertetraploid cells; B) chrysotile-treated cells recovered for 2 and 4 days show an increase in the number of cells in metaphase and a decrease in cells in anaphase and telophase compared with control cells (P<0.01 for 48 h and P<0.001 for 4 days).

Chrysotile-treated cells exhibited a 12% lower number of G1 cells compared with control cells, and also showed a 5% greater number of G2/M cells than did control cells (Table 3). These differences occurred regardless of the duration of the recovery period, and were more pronounced after longer periods of recovery. When the number of cells in S-phase was evaluated, no difference was observed between control and chrysotile-treated cells (Fig. 2, A).

Mitotic index of control and chrysotile-treated HK2 cells was quantified using immunofluorescence. Analysis of mitotic index showed that the total number of cells in M phase was similar in control and chrysotile-treated cells. However, in control cells, the number of cells in anaphase and telophase was greater than the number of cells in metaphase, while after 48 h of chrysotile treatment followed by 2 to 4 days of recovery, the number of cells in metaphase was greater than the number of cells in anaphase and telophase. After 8 days of recovery, the number of cells in metaphase was similar in chrysotile-treated and control cells (Fig. 2, B).

### HK2 and VERO cell morphology and presence of chrysotile fibers

Control and chrysotile-treated HK2 cells were analyzed by laser scanning confocal microscopy, after immunofluorescent labeling of microtubules, actin filaments and nuclei. Chrysotile fibers were visualized by their autofluorescence (see details in [13]). The presence of multinucleated and micronucleated cells, abnormal mitosis and fibers was analyzed and quantified.

**Figure 3:**
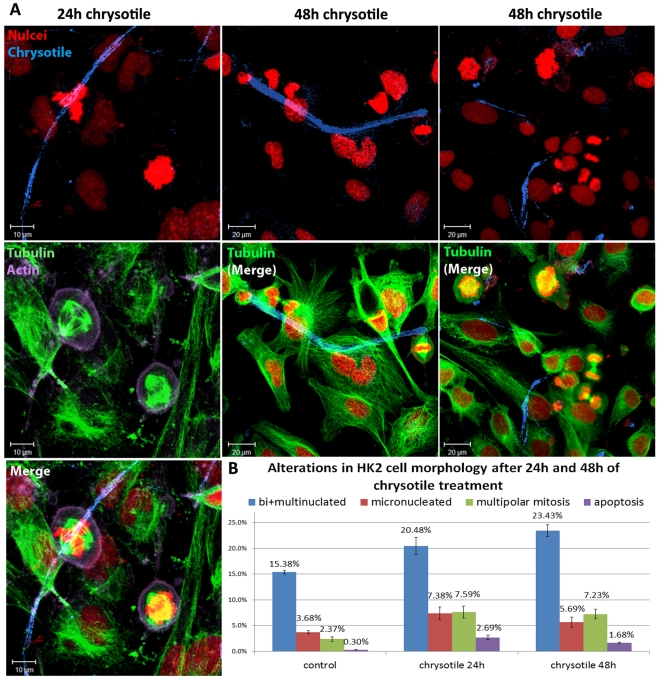
Alterations in the morphology of HK2 cells after 24 h or 48 h of chrysotile treatment. Cells were processed by immunofluorescence to visualize nuclei, actin filaments and microtubules, and chrysotile fibers were observed by their autofluorescence. A) Confocal images of HK2 cells treated with chrysotile for 24 h or 48 h showing long, thick fibers interacting with the cells and multipolar mitosis; B) the alterations in cell morphology were analyzed, and after 24 h or 48 h of chrysotile treatment, the number of binucleated and multinucleated cells increased, as well the number of micronucleated cells, apoptotic cells and cells in multipolar mitosis (P<0.001).

After 24 h and 48 h of chrysotile treatment, long fibers were observed interacting with the HK2 cell surface and actin filaments; some small fiber fragments were also detected inside cells (Fig. 3, A). Alterations in cell culture were observed, such as greater numbers of bi- and multi-nucleated, micronucleated and apoptotic cells. The number of cells with multipolar mitosis also increased, reaching 7.23% of all dividing cells after 48 h of chrysotile exposure (in control 2.37% of all cell divisions were multipolar) (Fig. 3, B).

**Figure 4:**
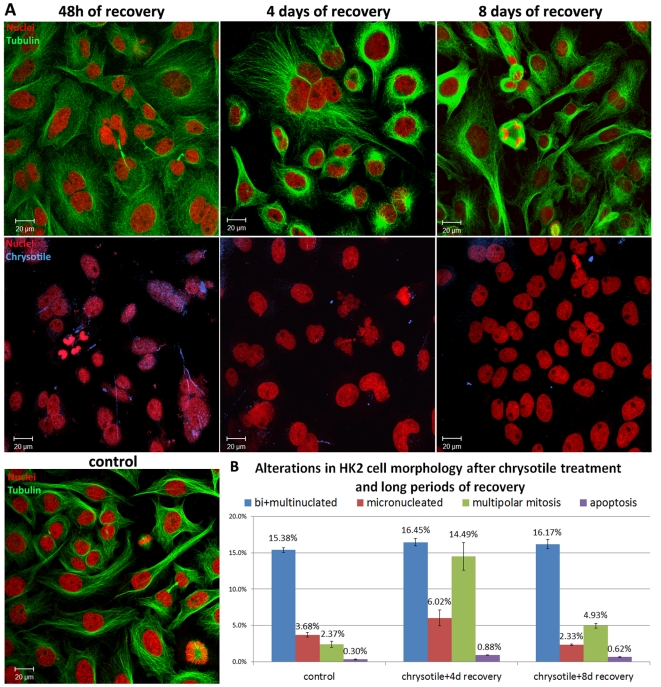
Alterations in the morphology of HK2 cells after 48 h of chrysotile treatment followed by 4 days and 8 days of recovery. Cells were examined using immunofluorescence to visualize nuclei, actin filaments and microtubules, and chrysotile fibers were observed by their autofluorescence. A) Confocal images of control HK2 cells and cells treated with chrysotile for 48 h and allowed to recover for 2 days, 4 days or 8 days, showing cell morphology and the presence of chrysotile fibers. After 2 days of recovery, long fibers were observed to interact with the cells surface; however, after 4 and 8 days of recovery, only fiber fragments were observed; B) the alterations in cell morphology were quantified, and after 48 h of chrysotile treatment and 4 days of recovery, the number of bi/multinucleated cells and apoptotic cells decreased, but was still higher than controls (P = 0.30 for bi/multinucleated after 4 days and P = 0.05 for apoptotic cells). The number of micronucleated cells in the chrysotile-treated group remained greater that in the control group (P<0.001 after 4 days), and the number of cells in multipolar mitosis was greatest (P<0.001). After 8 days of recovery, the number of cells in multipolar mitosis cells remained higher than in control cells (P = 0.02).

After 48 h of chrysotile treatment followed by a similar recovery period, multinucleated cells, abnormal mitosis and long and small chrysotile fibers inside cells were observed (Fig. 4, A). Further analysis showed that cells allowed to recover for long periods contained fewer fibers; chrysotile fibers and small fiber fragments were observed in the perinuclear region of cells after 4 days of recovery, while after 8 days of recovery there were no long fibers and few small fragments of fibers within cells (Fig. 4, A). After 4 days of recovery, the number of bi/multinucleated cells decreased but it was still higher than in control cells, however the numbers of cells in multipolar mitosis and micronucleated were increased (Fig. 4, B). After 8 days of recovery, the cell morphology of chrysotile-treated cells resembled that of control cells, which comprised mononucleated cells, cells in bipolar mitosis and few micronucleated and apoptotic cells. The number of cells in multipolar mitosis decreased compared with that of cells allowed to recover for 4 days; however, in the chrysotile-treated cells, there were more instances of multipolar mitosis than there were in the control cells (Fig. 4, B).

**Figure 5:**
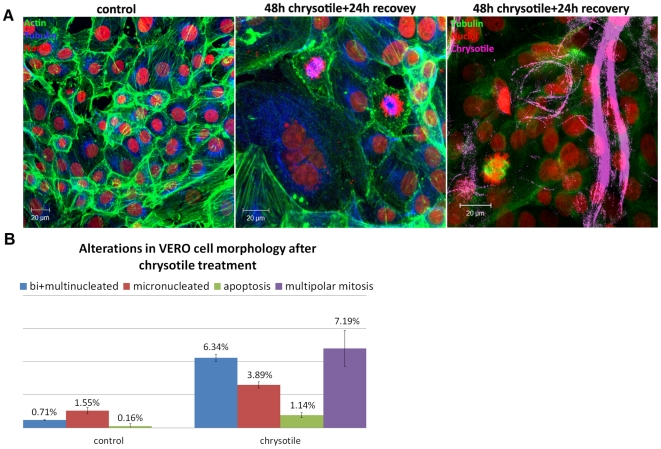
Alterations in VERO cell morphology after 48 h of chrysotile treatment and 24 h of recovery. Cells were examined by using submitted to immunofluorescence to visualize the nuclei, actin filaments and microtubules, and chrysotile fibers were observed by their autofluorescence. A) Confocal images of control cells and cells treated with chrysotile for 48 h and allowed to recover for 24 h, showing cell morphology and the presence of chrysotile fibers. After recovery, long fibers were observed to interact with the cells, and multinucleated cells, micronucleated cells and cells in multipolar mitosis were observed; B) the alterations in cell morphology were quantified, and chrysotile treatment led to increased numbers of micronucleated cells, bi/multinucleated cells, apoptotic cells and cells in multipolar mitosis (P<0.001).

To determine whether normal cells would similarly respond to chrysotile, cultures of VERO cells were exposed to chrysotile fibers for 48 h and allowed to recover for 24 h in fiber-free medium, and then they were processed using immunofluorescence to analyze cell morphology. The control cells comprised mainly mononucleated cells (99.29%), with rare micronucleated cells (1.55%) and no abnormal mitosis. After chrysotile treatment, bi/multinucleated and micronucleated cells were observed in greater numbers than in control cells (6.34% and 3.89% respectively), and multipolar mitosis and cytokinesis resulting in three daughter cells were also observed (7.18% of all cell divisions) (Fig. 5, A and B).

### Time-Lapse Microscopy: fates of abnormal mitotic cells and the formation of multiple centrosomes

**Figure 6:**
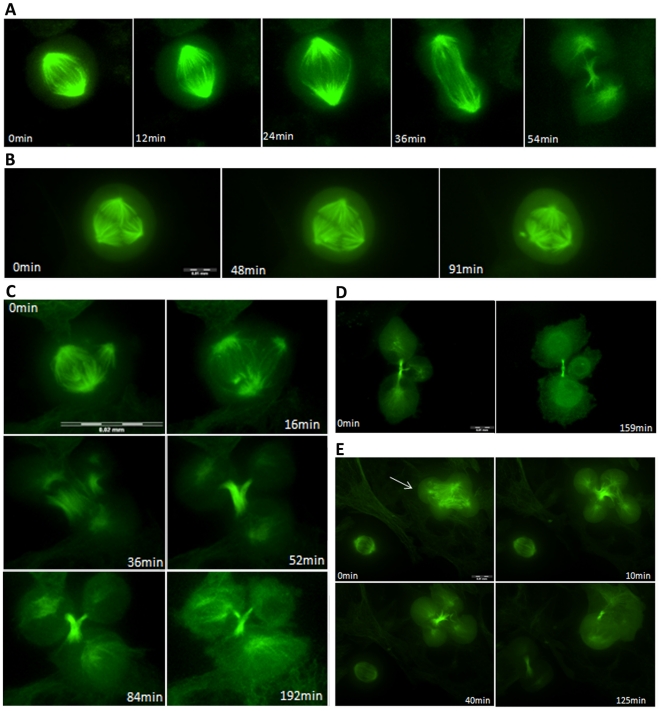
Fates of HK2 cells in multipolar mitosis after chrysotile treatment. Cells transfected with GFP-tagged α-tubulin were treated with chrysotile for 24 or 48 h and then observed by time-lapse spinning disk confocal microscopy. Cells in metaphase were observed for 2 to 3 h. A) A time series of maximal projection images showing a bipolar mitosis that generated only two daughter cells after 1 h in a control culture; B) a time series of maximal projection images showing a tripolar mitosis from a chrysotile-treated culture that did not progress through M phase; C) a time series of maximal projection images showing a chrysotile-treated cell that organized spindle poles in a tripolar fashion and progressed to anaphase and telophase generating three daughter cells; D) a time series of maximal projection images showing cytokinesis in a chrysotile-treated cell, generating three daughter cells that acquired interphase morphology; E) a time series of maximal projection images of a chrysotile-treated cell that entered anaphase generating four daughter cells; however, the cells merged during telophase and formed only two daughter cells.

For time-lapse spinning disk confocal microscopy, HK2 cells were transfected with GFP-tagged α-tubulin. Each transfected cell was observed for a period of 2–3 h. When a control cell in metaphase was found, it progressed to anaphase and reached telophase in 30 to 100 minutes. All the divisions observed in control cells were bipolar (Fig. 6, A). By contrast, when chrysotile-treated cells were observed, around 40% of metaphases were multipolar. Analysis of the fate of 30 of these cells revealed different patterns; each of them was observed at least twice.

About half of the cells in multipolar metaphase did not progress to anaphase during the observation period, remaining in metaphase with pseudo-bipolar, tripolar or quadripolar spindles (Fig. 6, B). In these cases, the microtubules were dynamic, and the spindle poles were more dynamic when grouped in pseudo-bipolar spindles. Cells in multipolar metaphase also underwent cell death, as evidenced by the leakage of the cytoplasm observed in the transmitted light channel.

Some cells in multipolar metaphase after chrysotile treatment also progressed to anaphase, telophase and cytokinesis. Tripolar metaphases generated three daughter cells linked by two midbodies with microtubule organization similar to that observed in control cells (Fig. 6, C). The three daughter cells either remained separated and acquired an interphase morphology 100 minutes after the beginning of cytokinesis (Fig. 6, D), or fused during cytokinesis. In these cases, two of the three daughter cells were fused and linked to the intercellular bridge by two structures of microtubules. The cell fusion also occurred during telophase until intercellular bridge formation, so two daughter cells were linked by only one midbody (Fig. 6, E).

The formation of multipolar spindles or multiple centrosomes was not observed during mitosis; all instances of metaphase were abnormal since the beginning of the observation. Thus, interphase cells were observed to identify alterations that could be related to centrosome amplification, which was responsible for the formation of multipolar spindles in the subsequent M phase.

**Figure 7:**
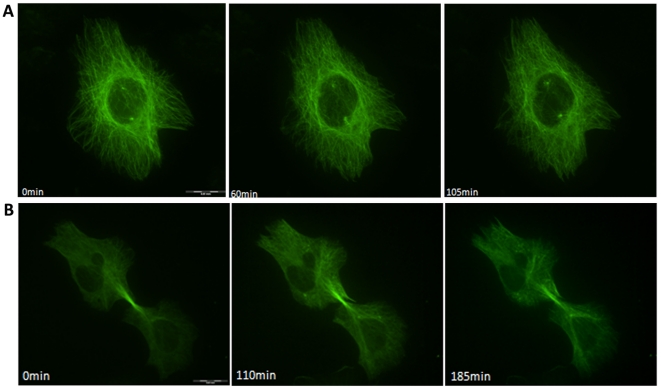
Alterations in interphase cells related to the formation of an abnormal number of centrosome. Alterations that could be related to an abnormal number of centrosomes were observed in HK2 cells treated with chrysotile for 24 or 48 h. A) A time series of images showing a cell with two centrosome like-structures that did not progress to M phase as expected for a cell with two centrosomes; B) a time series of images showing two interphase cells linked by an intercellular bridge without a midbody organization. The cells approached each other during the period of observation, reflecting a regression of cytokinesis.

Cells in interphase with two centrosomes were observed, and the centrosomes approached each other instead of migrating to opposite poles. Also, the microtubule network of these cells remained similar to interphase cells and did not form spindles. Another situation that was observed after chrysotile treatment was the presence of interphase with two centrosome-like bodies – structures located in perinuclear region of the cell where microtubules were concentrated. These structures appeared to be formed by very small dots that moved during all the recording period. Also, the cell remained in interphase and did not progress to M phase (Fig. 7, A).

Another mechanism responsible for the formation of extra centrosomes in cells is cytokinesis failure. This process takes too long to be observed during the course of the time-lapse experiments we conducted. However, interphase cells linked by an intercellular bridge without a midbody microtubule organization were observed, and the cells approached each other during the observation period (Fig. 7, B).

## Discussion

Chrysotile is considered less harmful to human health than others types of asbestos, due to the lack of a strong association between chrysotile fiber exposure and the development of carcinomas and mesotheliomas. Nevertheless, some studies have shown the potential of chrysotile to cause DNA damage and cellular alterations *in vitro*, and lung injuries *in vivo*. The present work analyzed cellular alterations related to aneuploidy and cell cycle disruption, and verified which alterations persist in culture after 2, 4 and 8 days of recovery in fiber-free medium. Also, alterations in cell morphology were related to the presence of fibers in culture during the first 24 h and 48 h of chrysotile treatment and also after long periods of recovery. We also analyzed multipolar mitosis and centrosome amplification, which are additional features of chrysotile treatment that are closely related to aneuploidy.

The analysis of HK2 cells by confocal microscopy reveled that chrysotile fibers did not persist in cell culture after 8 days of recovery, and after 4 days of recovery only fragments and small fibers were present in the perinuclear region of some cells. Also, after 4 and 8 days of recovery, cell morphology resembled that of control cells with respect to the presence of bi/multinucleated and micronucleated cells. However, the aneuploid population persisted in cell culture after 4 and 8 days of recovery in fiber-free medium, and the percentages of aneuploid nuclei were always around 10% of the total population. The data provided by DNA quantification also showed that aneuploid nuclei were mostly in mononucleated cells after 4 and 8 days of recovery, while during the first days of recovery the aneuploid nuclei were in bi/multinucleated cells. All these observations are in agreement with the data provided by flow cytometry, which indicated that chrysotile-treated HK2 cells showed increased hipertetraploidy even after 8 days of recovery.

The presence of aneuploid cells is a consequence of chrysotile treatment observed after 48 h of exposure, which persists even after 72 h of recovery following the treatment [11], [13]. These cells could be related to cancer development, because the loss or gain of one chromosome - or a portion thereof - can introduce mutations required for malignant transformation. We show in this study that the induction of aneuploidy in cells is chrysotile concentration-dependent, and as described above, that the percentage of aneuploid cells remained high compared with control cells after up to 8 days of recovery in fiber-free culture medium. These aneuploid cells can persist in culture if they are able to progress through and finish the cell cycle, or if chrysotile fibers continue in cell culture, thereby inducing new aneuploid cells during recovery. Since just a few fiber fragments were observed in cell culture after 4 and 8 days of recovery, new aneuploid cells could have been induced by the residual fiber fragments. However, it is unlikely that the residual fiber fragments would have induced a similar extent of aneuploidy as did during the first hours of treatment. Thus, to maintain the frequency of aneuploid cells in culture, the cells likely progressed through the cell cycle and generated new aneuploid cells.

Multipolar mitosis is linked to aneuploid cell formation because of abnormal chromosome segregation, which is caused by cell divisions resulting in more than two cells and erroneous attachment between kinetochores and microtubules. In the present study, time-lapse experiments allowed the analysis of the fate of HK2 cells in the multipolar mitosis that is induced by chrysotile treatment. Half of the cells in chrysotile-induced multipolar metaphase did not progress through mitosis and some could undergo cell death, the other half finished the cell cycle, generating two or three daughter cells. When a daughter cell was generated by cell fusion during telophase or cytokinesis that cell (likely aneuploid) had more than one centrosome and in the next M phase underwent a new multipolar mitosis. However, when a multipolar mitosis generated three daughter cells, these cells were mononucleated, showing one centrosome, and might be aneuploid, leading to increased number of mononucleated, aneuploid cells.

Chrysotile-treated HK2 cells showed high number of cell in multipolar mitosis after 48 h of treatment and after up to 8 days of recovery. Initially, the cells with extra centrosomes could be formed by either chrysotile interference, leading to centrosome amplification or fragmentation, or after cytokinesis failure, generating one interphase tetraploid cell with two centrosomes. The process of centrosome amplification is now known to be affected by oxidative stress and DNA damage [27], [28], and it also occurs after treatment of cells with drugs, such as AZT and hydroxyurea, and after alterations in cell cycle and expression levels of cyclin [29], [30], [31]. Some studies have shown that chrysotile treatment leads to oxidative stress, DNA damage and cell cycle disruptions, processes that can lead to centrosome amplification. Also, the regression of cytokinesis following asbestos exposure has been described [14], generating cells with two centrosomes.

In the present study, we verified that the number of cells in multipolar mitosis decreased after 48 h of chrysotile treatment followed by 8 days of recovery, but remained greater than in control cells. The mechanism involved in centrosome amplification after chrysotile exposure might decrease in the absence of fibers after the long period of recovery. The greater number of cells in multipolar mitosis could arise from previous multipolar mitosis as discussed above, when multipolar mitosis could generate cells with and without abnormal numbers of centrosomes, reducing the frequency of cells with extra centrosomes after a few divisions.

Chrysotile treatment also led to an increased percentage of cells in G2/M. These cells might be arrested in G2 because problems in DNA replication are detected at the G2/M checkpoint, or during M phase, due to difficulties in chromosome alignment and kinetochore-to-microtubule attachment. Mitotic indices analyzed after chrysotile treatment demonstrated an increased number of cells in metaphase, and a decreased number of cells in anaphase and telophase. These data indicated that metaphase could last longer after chrysotile treatment than it does in control cells, and this delay could occur as a consequence of alterations in chromosome alignment. In time-lapse experiments, half of the cells in multipolar metaphase remained in metaphase during the course of the experiment, demonstrating that multipolar metaphase can last longer than bipolar mitosis.

Some authors have demonstrated that cells with compromised (or weakened) checkpoint machinery can progress to anaphase with one or more chromosomes unattached to microtubules, and these cells can arrest in the cell cycle but eventually finish the cell cycle, generating aneuploid cells [32], [33]. The cells with weakened checkpoint can generate chromosomal instability when a few chromosomes are not correctly attached to microtubules, and the cells complete metaphase, thus generating aneuploid cells [32]. These kind of abnormality could be generated by alterations in checkpoint genes (Bub1, BubR1, Bub3, Mad2) such as the heterozygozity observed both in human cancer cells and patients with the rare recessive disorder mosaic variegated aneuploidy [34], [35].

Multipolar mitosis also leads to erroneous microtubule-kinetochore attachment, and cells with a weakened mitotic checkpoint can progress to anaphase even with abnormal attachments. Time-lapse experiments with chrysotile-treated cells showed that these events can occur in the HK2 cells used in the present work, which are tumor cells and probably have mutations that allow progression of the cell cycle even after abnormal mitosis.

The use of normal epithelial lung cells would be interesting to compare with the response to chrysotile treatment in genetically normal and abnormal cells. However, there is no normal epithelial lung cell line established. The use of VERO cells (normal epithelial cell line) demonstrated that the morphological alterations detected in HK2 cells, such as multipolar mitosis and micronucleated cells, were also detected in normal cells, indicating that some chrysotile-responses did not depend on the genetic stability.

Cell cycle analysis by flow cytometry revealed that chrysotile-treatment led to decreased percentages of cells in G0/G1, but did not lead to an increase of cells in S phase, indicating that the treated cells enter the cell cycle similarly to control cells. Mitotic indices also demonstrated similar numbers of mitotic cells in control and chrysotile-treated cells. Therefore, chrysotile treatment did not increase proliferation in cultures HK2 cells.

*In vivo* experiments indicated that chrysotile exposure affects proliferation in lung epithelium and mesenchymal cells *in vivo* [36], [37], [38]. This proliferative effect appears to be a response to lung injury caused by fibers and persist 6 months after 3 days of exposure to repair the epithelium and extra-cellular matrix. However, in the current *in vitro* study, no proliferative effects were induced by chrysotile exposure. The proliferative response observed *in vivo* after chrysotile exposure involves the interaction of different cell types present in lung tissue, as well as the extracellular matrix and the production of many growth factors that regulate the proliferation of cells proximal to the injured tissue; such interactions are absent *in vitro*.

The mutagenic effect of chrysotile exposure was detected by the micronucleus assay. In this study, chrysotile treatment led to formation of micronuclei, consistent with previous reports [11]. However, after 8 days of recovery in fiber-free medium, the percentage of micronucleated cells decreased and was similar to that in control cells. These findings indicate that the potential of chrysotile to cause DNA damage, such as double strand breaks and mitotic dysfunctions leading to formation of micronuclei, does not persist after long periods of recovery. If the residual chrysotile present in cell culture after 8 days of recovery is not sufficient to induce the formation of micronuclei, perhaps all alterations that persist in cell culture after long periods of recovery are a consequence of the initial alterations caused by chrysotile, which remain after subsequent cell divisions and are not the direct action of chrysotile.

In summary, the present work showed that HK2 cells exposed to chrysotile fibers for 48 h followed by 2 and 4 days of recovery exhibited alterations that were not observed in control HK2 cells. These alterations included increased numbers of aneuploid cells, decreased numbers of G1 cells and increased numbers of G2/M cells, micronucleated cells, cells in early M phase and cells in multipolar mitosis. The aneuploid population and presence of multipolar mitosis persisted in cell culture up to 8 days after treatment, when only a few chrysotile fragments remained, and cell morphology was similar to that of control cells. During the treatment and in the first 2 days of recovery, chrysotile caused DNA and cell damage, leading to formation of micronuclei, amplification of centrosomes and disruptions of mitosis that can form multinucleated and aneuploid cells. Some cells in multipolar mitosis are able to progress through the cell cycle and form new (probably aneuploid) cells, of uncertain viability. However, some of the aneuploid cells generated were viable, as aneuploidy persisted in the cultures after long recovery periods and in the absence of chrysotile fibers.
